# Autophagy augments the self-renewal of lung cancer stem cells by the degradation of ubiquitinated p53

**DOI:** 10.1038/s41419-021-03392-6

**Published:** 2021-01-19

**Authors:** Jianyu Wang, Doudou Liu, Zhiwei Sun, Ting Ye, Jingyuan Li, Bin Zeng, Qiting Zhao, H. Rosie Xing

**Affiliations:** 1 Institute of Life Sciences, Chongqing Medical University, Chongqing, China; 2 State Key Laboratory of Ultrasound Engineering in Medicine Co-Founded by Chongqing and the Ministry of Science and Technology, School of Biomedical Engineering, Chongqing Medical University, Chongqing, China

**Keywords:** Cancer stem cells, Cancer stem cells

## Abstract

It has been postulated that cancer stem cells (CSCs) are involved in all aspects of human cancer, although the mechanisms governing the regulation of CSC self-renewal in the cancer state remain poorly defined. In the literature, both the pro- and anti-oncogenic activities of autophagy have been demonstrated and are context-dependent. Mounting evidence has shown augmentation of CSC stemness by autophagy, yet mechanistic characterization and understanding are lacking. In the present study, by generating stable human lung CSC cell lines with the wild-type TP53 (A549), as well as cell lines in which TP53 was deleted (H1229), we show, for the first time, that autophagy augments the stemness of lung CSCs by degrading ubiquitinated p53. Furthermore, Zeb1 is required for TP53 regulation of CSC self-renewal. Moreover, TCGA data mining and analysis show that Atg5 and Zeb1 are poor prognostic markers of lung cancer. In summary, this study has elucidated a new CSC-based mechanism underlying the oncogenic activity of autophagy and the tumor suppressor activity of p53 in cancer, i.e., CSCs can exploit the autophagy-p53-Zeb1 axis for self-renewal, oncogenesis, and progression.

## Introduction

Despite improved treatment options for lung cancer, its morbidity and mortality rate remain the highest among all solid tumors^1^. Late detection and presentation, resistance to therapies, aggressive metastasis, and frequent recurrence are the main reasons for its poor clinical prognosis^2^. Although the involvement of cancer stem cells (CSCs) in all aspects of human cancer has been postulated, the mechanisms governing the regulation of CSC self-renewal in cancer state are poorly defined.

Mounting evidence has shown that autophagy may promote the stemness of CSCs^3–5^. Autophagy is an evolutionarily conserved biological process responsible for energy metabolism for the maintenance of homeostasis under nutrient-deprived or other stressful conditions^6^. Both pro- and anti-oncogenic activities of autophagy have been reported and are context-dependent^7,8^. On the one hand, autophagy can inhibit malignant transformation by preventing the accumulation of damaged proteins, organelles, and mitochondria^9^. On the other hand, the highest autophagy activity is found in areas of cancer cell aggregates where nutritional needs are increased and they may be nutrient-deprived^10^. Autophagy promotes the survival of cancer cells by providing biochemical reaction substrates derived from the degradation of intracellular organelles and proteins. During the initial stage of metastasis, autophagy may inhibit metastasis by increasing the release of anti-metastatic immunomodulatory factors. Once tumor cells enter hematogenous circulation, autophagy may augment metastasis by protecting the circulating tumor cells from anoikis. During colonization at the metastatic site, the role of autophagy becomes more intricate. Autophagy keeps the extravasated tumor cells in the dormancy stage, thus preventing proliferation and colonization. Once micro-metastases are established, autophagy switches to promote the proliferation of macro-metastases by helping tumor cells adapt to the stressful foreign microenvironment. Furthermore, emerging experimental evidence has demonstrated that the pro-oncogenesis and metastatic activity of autophagy may be achieved by augmenting the stemness of CSCs^11–13^. However, the mechanistic understanding underlying the regulation of CSC self-renewal by autophagy is questionable and limited.

TP53, the most well-characterized tumor suppressor, can activate or inhibit autophagy depending on its intracellular localization. Nuclear localized p53 activates autophagy via transcriptional activation of key autophagy-related genes, such as sestrin^14–16^. In contrast, cytosolic p53 inhibits autophagy via AMPK and mTOR^17^. Recent studies have shown that p53 degradation is subjected to autophagy regulation, where mitochondria-associated p53 is degraded by mitophagy^18^ and acetylated p53 is degraded by autophagy, including the mutant p53^17,19–23^. The recently reported role of TP53 in the regulation of CSC stemness requires validation and mechanistic investigation^18,24,25^. In addition, p53 could also activate miR-200 and miR-34 directly^26–29^, which could inhibit the epithelial–mesenchymal transcription factors (EMT TFs) such as Zeb1, Snail1, and Twist2^30–33^. These EMT TFs have been proven to be the key regulatory factors in regulating the self-renewal of CSCs^13,34–37^.

In this study, by generating stable human lung CSC cell lines with the wild-type TP53 (A549), and cell lines where TP53 has been deleted (H1229), we show, for the first time, that autophagy augments the stemness of lung CSCs by degrading ubiquitinated p53, thus relieving the inhibition of cytosolic p53 on autophagy. Furthermore, Zeb1 is required for p53 regulation of CSC self-renewal. Moreover, The Cancer Genome Atlas (TCGA) data mining and analysis show that Atg5 and Zeb1 are poor prognostic markers of lung cancer. In summary, the present study has uncovered a new mechanism underlying the oncogenic activity of autophagy in that autophagy augments lung CSC stemness through degradation of tumor suppressor p53.

## Materials and methods

### Animals

BALB/cA-nude nude mice were purchased from the Experimental Animal Centre of Chongqing Medical University.

### Compliance with ethics guidelines

Animal studies were conducted in accordance with an approved protocol and with the institutional animal welfare guidelines of the Chongqing Medical University.

### Cell culture

A549 and H1299 human lung cancer cell lines were obtained from the Stem Cell Bank of the Chinese Academy of Sciences. Cells were cultured with Dulbecco’s modified Eagle medium (DMEM) supplemented with 1% amphotericin B, 1% penicillin–streptomycin, and 10% fetal bovine serum. A549 and H1299 CSC derivative cell lines, the A549-oncosphere, and the H1299-oncosphere were generated as previously described^38^. Cells were cultured in the DMEM/F12 supplemented with 2% B27, 1% amphotericin B, and 1% penicillin–streptomycin, with 20 ng/ml epidermal growth factor (Beyotime) and 20 ng/ml fibroblast growth factor (Beyotime).

### Reagents for inhibiting and inducing autophagy

3-MA (M9281) was purchased from Sigma-Aldrich; BafA1 (S1413), rapa (S1039), and NVP-CGM097 (S7875) were purchased from Selleck; and Mdivi-1 (HY-15886) and CCCP (HY-100941) were purchased from Master of Small Molecules.

### Six-well plate serial spheroid formation assay

Single-cell suspensions were plated at 1000 cells/well in six-well plates. After every 2 weeks in culture, clonogenic spheroids containing >50 cells were counted under microscopy. Spheroid cultures were then collected, and single-cell suspensions were prepared for setting up the second round of the assay. The assay was repeated for three consecutive rounds.

### 96-well plate single-cell cloning assay

A single-cell suspension was prepared and the concentration was adjusted to 10 cells/ml. One hundred microliters of cell suspensions were seeded into each well of 96-well plates (ThermoFisher Scientific, USA). Single-cell seeding in each well was confirmed using microscope and wells containing one cell were marked. After being cultured at 37 °C with 5% CO_2_ for 10 days, colonies exceeding 50 cells were counted.

### Subcutaneous tumor transplantation assay in BALB/c nude mice

The 1 × 10^3^ single-cell suspensions were mixed with 50 μL Matrigel Matrix (Corning) at a 1:1 ratio. Then, 100 μL of mixture was injected subcutaneously into both insides of the hind legs of BALB/c nude mice. Tumor size was measured every 2 days and tumor volume was calculated using *V* = (length x width^2^)/2. Mice were euthanized when tumor volume reached ~1000 mm^3 38^.

### Reverse transcription and quantitative real-time polymerase chain reaction

Total RNA was isolated per the protocol of Eastep^®^ Super Total RNA Extraction Kit (Promega). Reverse transcription-polymerase chain reaction (RT-PCR) was conducted using PrimeScript RT Master Mix (Takara) and quantitative polymerase chain reaction (q-PCR) was conducted using PrimeScript^™^ RT Master Mix (Takara) according to the manufacturer’s instructions. Relative expression was normalized to the internal control, TATA binding protein (Tbp). In a 10-µl reaction volume, the following PCR cycling parameters were used in the Light Cycler: 39 cycles of 95 °C for 30 s, 95 °C for 5 s, followed by 60 °C for 30 s. PCR primer sequences are listed in Table 1.

**Table 1:** PCR primer sequence.

Gene name	Forward primers	Reverse primers
*Oct4*	CGAAAGAGAAAGCGAACCAG	TGAAGTGAGGGCTCCCATAG
*Nanog*	AGATGCCTCACACGGAGACT	TCTGGAACCAGGTCTTCACC
*Aldh1*	TGGACCAGTGCAGCAAATCA	CGCCATAGCAATTCACCCAC
*CD133*	CCCCGCAGGAGTGAATCTTT	GAAGGACTCGTTGCTGGTGA
*ATG5*	TGCAGATGGACAGTTGCACA	CCACTGCAGAGGTGTTTCCA
*TP53*	TGTGACTTGCACGTACTCCC	ACCATCGCTATCTGAGCAGC
*Zeb1*	GAGGAAGAGGAGGAGGAGGA	GCTTGACTTTCAGCCCTGTC
*HDM2*	TCTTGATGCTGGTGTATATCAAGT	AATTCTCACGAAGGGCCCAA
Tbp	CCACAGCTCTTCCACTCACA	CTGCGGTACAATCCCAGAAC
miR-34a-5p	CTGGCAGTGTCTTAGCTGGTTGT	
miR-200C	CCGTAATACTGCCGGGTAATGATGGA	

### Packaging of shRNA lentivirus

The small interfering RNA (siRNA) of HDM2 [GCTGGTGTAAGTGAACATT (5′–3’) and UUCGACCACACATTCACTTGT (5′–3′)] was purchased from GenePharma. Plasmid construction and lentivirus packaging were also prepared and supplied by GenePharma. Lentivirus-mediated short hairpin RNA (LV-shRNA) was from H1/GFP&Puro and LV-OE was from EF1a/GFP&Puro.

### Western blot (WB) analysis

WB was conducted as previously reported^34^. In brief, cells were lysed in radioimmunoprecipitation assay buffer (Beyotime, China) containing 1% phenylmethylsulfonyl fluoride. Extracted protein concentration was measured by the bicinchoninic acid method and stored at −80 °C. A 25-µg protein sample was run on an 8–10% sodium dodecyl sulfate–polyacrylamide gel electrophoresis (SDS-PAGE) gel and transferred to a polyvinylidene difluoride membrane (Bio-Rad, USA). Afterward, the membrane was blocked in 5% nonfat milk (BD, USA) and incubated with the primary antibodies at 4 °C overnight. Finally, the membrane was incubated with the secondary antibodies and developed under the gel electrophoresis imager (Bio-Rad, USA). The primary antibodies of LC3 (ab192890), P62 (ab207305), and ATG5 (ab109490) were purchased from Abcam, while p53 (10442-1-AP), Zeb1 (21544-1-AP), and GAPDH (10494-1-AP) antibodies were purchased from Proteintech. The secondary antibody anti-Rabbit (SA00001-2) was also purchased from Proteintech.

### Immunofluorescence analysis of LC3 puncta and asymmetric CSC cell division

Quantification of RFP-LC3 puncta was conducted by infecting cells with the adenovirus-expressing RFP-LC3B fusion protein (Beyotime, C3011). The percentage of cells showing accumulation of RFP-LC3 puncta was counted and quantified. The BrdU assay for assessing the symmetric cell division (SD) and asymmetric cell division (ASD) was conducted as previously described^38^. In brief, cells were treated with BrdU for a minimum of seven days. Following treatment, BrdU was removed for at least two days and cells were fixed with ice-cold 70% ethanol and treated with hydrochloric acid. Thereafter, cells were incubated with the primary antibody (anti-BrdU, Millipore-Sigma) at 4 °C for 12 h, followed by incubation with the secondary antibody (Proteintech) at 37 °C for 1 h. Images were taken by an OLYMPUS inverted fluorescence microscope.

### Analysis of autophosome by transmission electron microscopy (TEM)

TEM was conducted as previously reported^39^. In brief, cells were harvested and centrifuged at 1200 rpm/min. The cell pellet was fixed with 4% glutaraldehyde for 2 h at 4 °C and fixed with 1% osmium tetroxide for 1 h at 4 °C. Next, cells were dehydrated in a graded series of alcohol and acetone, followed by embedment in Epon 816 (Electron Microscopy Sciences, Hatfield, PA, USA). Ultrathin sections were cut by a Leica ultramicrotome (Leica Microsystems, Buffalo Grove, IL, USA) and stained with uranyl acetate and lead citrate. TEM was conducted by a JEM-1400Plus transmission electron microscope (JEOL Ltd. Tokyo, Japan).

### TCGA data analysis

mRNA expression of Atg5 and Zeb1 in human lung adenocarcinoma was analyzed by TCGA Research Network (http://cancergenome.nih.gov). To analyze the survival of patients with lung adenocarcinoma, patient samples were analyzed by OncoLnc (http://www.oncolnc.org).

### Statistical analysis

Data were analyzed by a parametric test using GraphPad Prism software and presented as the mean ± SD. Differences were considered statistically significant when *P* < 0.05(*) and *P* < 0.01(***) and different superscripts represent significant difference, *p* < 0.05.

## Results

### Generation and characterization of A549 and H1299 derived CSC lines

**Fig. 1:**
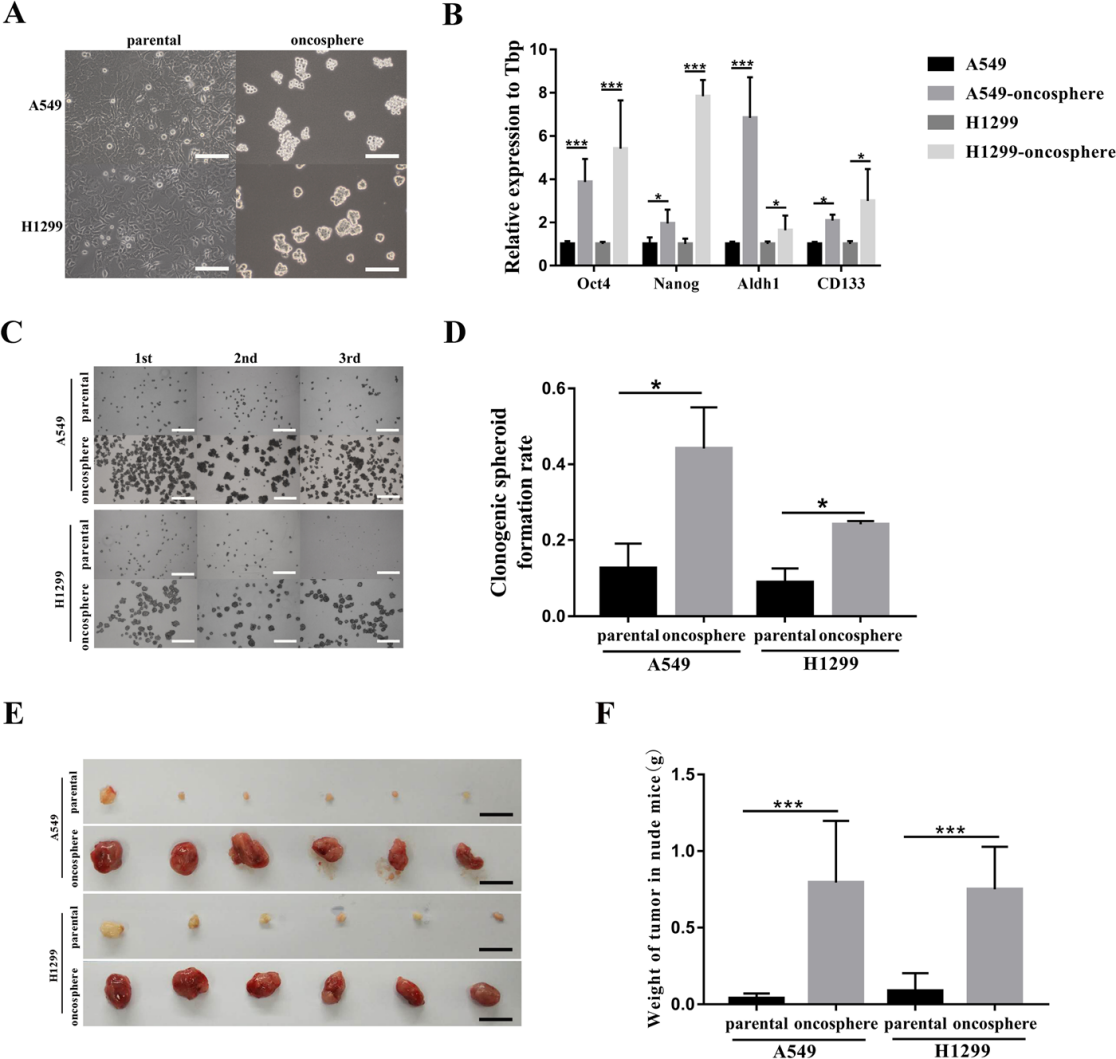
Generation and characterization of CSC cell lines derived from A549 and H1299 parental cell lines. **A** The morphology of A549, A549-oncosphere, H1299, and H1299-oncosphere, bar = 120 µm. **B** Analysis of mRNA expression of Oct4, Nanog, Aldh1, and CD133. Tbp expression was used as a reference control. *p < 0.05, ***p < 0.01. **C** Consecutive spheroid formation assay, 1st means single-cell suspensions were plated at 1000 cells/well, 2nd means spheroid cultures were then collected from 1st and single-cell suspensions were prepared for setting up the second round of the assay at 1000 cells/well, and so forth. The 3rd means 1000 cells come from the 2nd, bar = 120 µm. **D** Single-cell cloning assay, 100 µl of 10 cells/ml concentration cell suspensions was seeded into each well of 96-well plates. After 10 days, colonies exceeding 50 cells were counted. *p < 0.05. **E** Tumors resected from nude mice inoculated with A549, A549-oncosphere, H1299, and H1299-oncosphere, respectively, bar = 1 cm. **F** Tumor weight in mice of which 10^3^ parental and oncospheres from the A549 and H1299 were injected. ***p < 0.01.

To investigate mechanisms underlying autophagy regulation of CSC self-renewal, the CSC component in A549 and H1299 cell lines was obtained by enriching the floating spheroids in defined stem cell media, a method we have developed and previously described^38^. The resulting CSC cell lines were named A549-oncosphere and H1299-oncosphere, respectively (Fig. 1A). Significant enhancement of stemness features of the A549- and H1299-oncospheres was confirmed in vitro by (1) the increased expression of stem markers, including Oct4, Nanog, Aldh1, and CD133 (Fig. 1B); and (2) enhanced self-renewal measured by the serial spheroid formation assay and the single-cell cloning assay (Fig. 1C, D). In vivo, 10^3^ cells of A549 and H1299 parental and CSC cell lines were injected subcutaneously into nude mice and the tumors were excised and weighed on day 21 post inoculation. Tumors derived from A549- and H1299-oncospheres were significantly bigger and heavier than those of A549 and H1299 parental cells (Fig. 1E, F). Collectively, both in vitro and in vivo characterization have confirmed that A549- and H1299-oncosphere cells possess the stemness features of CSCs and are more oncogenic.

### Autophagy augments the stemness only in A549-oncosphere cells, but not the H1299-oncosphere cells

**Fig. 2:**
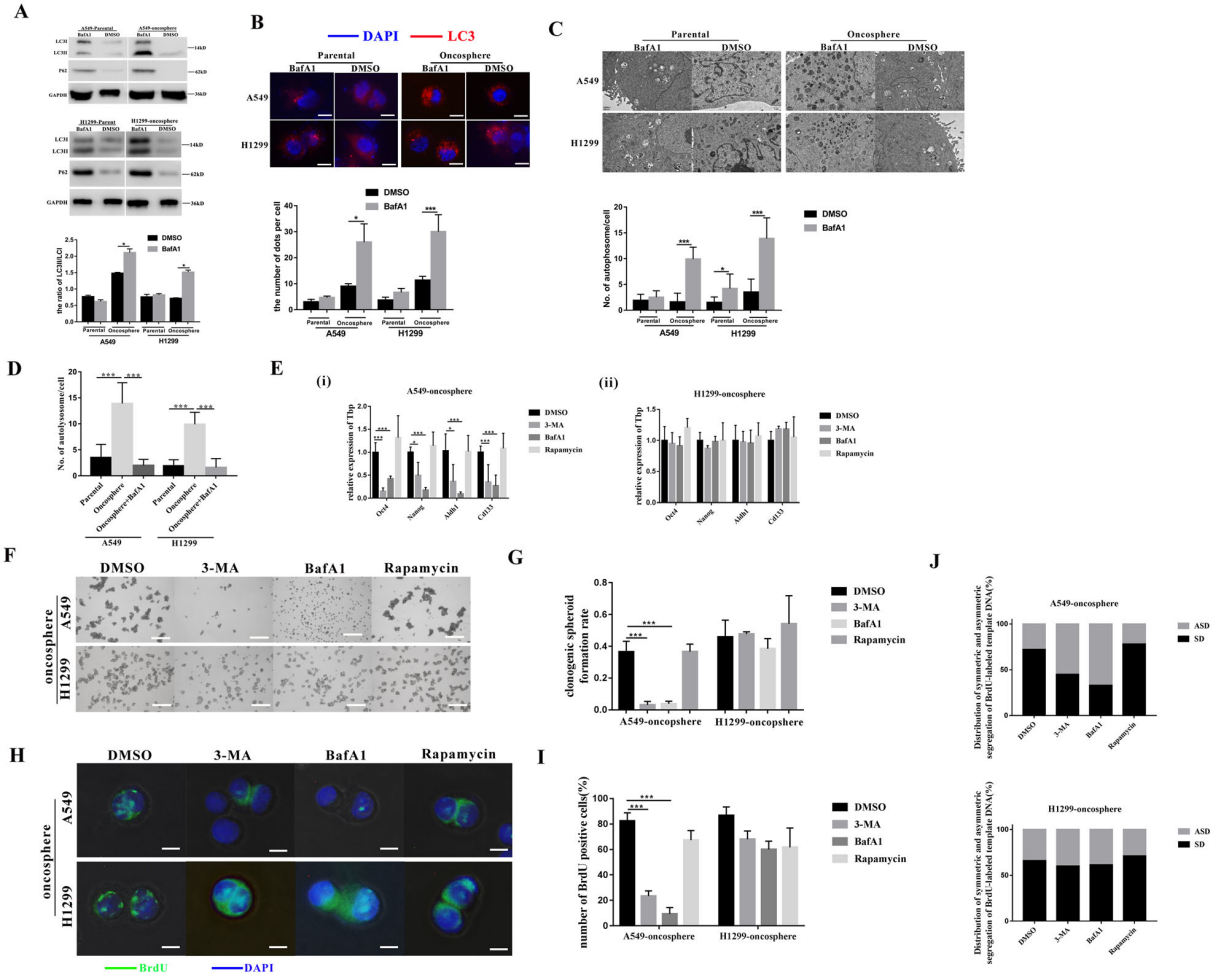
Autophagy augments the stemness of A549-oncosphere in vitro. **A** Analysis of protein levels of LC3 and P62 in A549, A549 + BafA1, A549-oncosphere, A549-oncosphere+BafA1, H1299, H1299 + BafA1, H1299-oncosphere, and H1299-oncosphere+BafA1 by western blot (WB), GAPDH was used as a reference control. **B** Immunofluorescent staining of RFP-LC3 in A549, A549 + BafA1, A549-oncosphere, A549-oncosphere+BafA1, H1299, H1299 + BafA1, H1299-oncosphere, and H1299-oncosphere+BafA1; the red bar refers to LC3 and the blue bar refers to DAPI; white bar = 30 µm. **C** TEM analysis of autolysosomes in A549, A549 + BafA1, A549-oncosphere, A549-oncosphere+BafA1, H1299, H1299 + BafA1, H1299-oncosphere, and H1299-oncosphere+BafA1; bar = 1 µm. **D** Quantification of autolysosome in A549, A549-oncosphere, A549-oncosphere+BafA1, H1299, H1299-oncosphere, and H1299-oncosphere+BafA1. ***p < 0.01. **E** (i) and (ii) mRNA expression of Oct4, Nanog, Aldh1, and CD133 in A549-oncosphere and H1299-oncosphere treated with DMSO, 3-MA, BafA1, and rapa. Tbp was used as a reference control. *p < 0.05, ***p < 0.01. Colony formation assay (**F**, bar = 120 µm) and single-cell cloning assay (**G**, ***p < 0.01.) using A549-oncosphere and H1299-oncosphere treated with DMSO, 3-MA, BafA1, and rapa. **H** Image of BrdU immunofluorescence; the green bar refers to BrdU and the blue bar refers to DAPI (**H**, white bar = 30 µm); and quantification of the number of BrdU-positive cells (**I**, ***p < 0.01) in A549-oncosphere and H1299-oncosphere treated with DMSO, 3-MA, BafA1, and rapa. **J** Distribution of symmetric and asymmetric segregation of BrdU-labeled template DNA in A549-oncosphere and H1299-oncosphere treated with DMSO, 3-MA, BafA1, and rapa. The concentration of 3-MA, BafA1, and rapa was 3 mM, 100 nM, and 5 µM, respectively. Cells used in this figure were treated for 24 h.

To investigate the involvement of autophagy in human lung CSC self-renewal, we first assessed the level of autophagy in parental and A549-oncosphere and H1299-oncosphere CSC cell lines. Autophagy was characterized by three classic assays commonly used in autophagy research: (1) the protein level of LC3 and P62 by WB, (2) RFP-LC3 punta by immunofluorescence (IF), and (3) TEM analysis of cytoplasmic accumulation of autophosomes. The increased ratio of LC3II/LC3I and decreased P62 expression (Fig. 2A), increased autophagic flow (number of puncta) (Fig. 2B), and cytoplasmic accumulation of autophosomes in the A549- and H1299-oncospheres (Fig. 2C, D) after BafA1 treatment collectively show that autophagic activity is higher than that of in the respective parental cell lines.

Next, we determined whether autophagy regulates the self-renewal of lung CSCs. Autophagy was inhibited by 3-MA and BafA1, which inhibit the early- and late-stage of autophagy, respectively, in A549- and H1299-oncospheres. Determined by stem cell gene expression (Oct4, Nanog, Aldh1, and CD133) in the six-well spheroid formation assay and the single-cell cloning assay, we found autophagy was only inhibited in A549-oncosphere. No significant changes in self-renewal were observed in the H1299-oncosphere (Fig. 2E–G). The differential response of the two lung CSC cell lines to changes in autophagy activity was confirmed by treatment with rapamycin (rapa), a stimulator of autophagy (Fig. 2E–G). The expression of the stemness gene was detected in A549- and H1299-oncospheres with added DMSO, 3-MA, BafA1, and rapa, via q-PCR. We found that mRNA levels were higher in the rapa group than the control group and there were no significant differences between inhibition of the autophagy group and control group (Fig. S1A, B). Recent studies, including our own, have shown that CSCs undergo SD and ASD, as normal stem cells do. Thus, we assessed changes in the BrdU retention rate and the SD/ASD ratio in A549- and H1299-oncospheres upon autophagy inhibition by 3-MA and BafA1, or activation by rapa. Interestingly, a reduced number of BrdU-positive cells and decreased ratio of SD/ASD were detected in A549-oncosphere cells when autophagy was inhibited, while these changes were absent in H1299-oncosphere cells (Fig. 2H–J). It is likely due to the elevated autophagy activity in A549- and H1299-oncosphere that has already maximally augmented the stemness features (Fig. 2A–E); further activation of autophagy by rapa produced no further augmentation of the stemness in A549-oncosphere cells (Fig. 2H–J).

### The stemness of A549-oncosphere, not that of the H1299-oncosphere, is inhibited by ATG5 knockdown

**Fig. 3:**
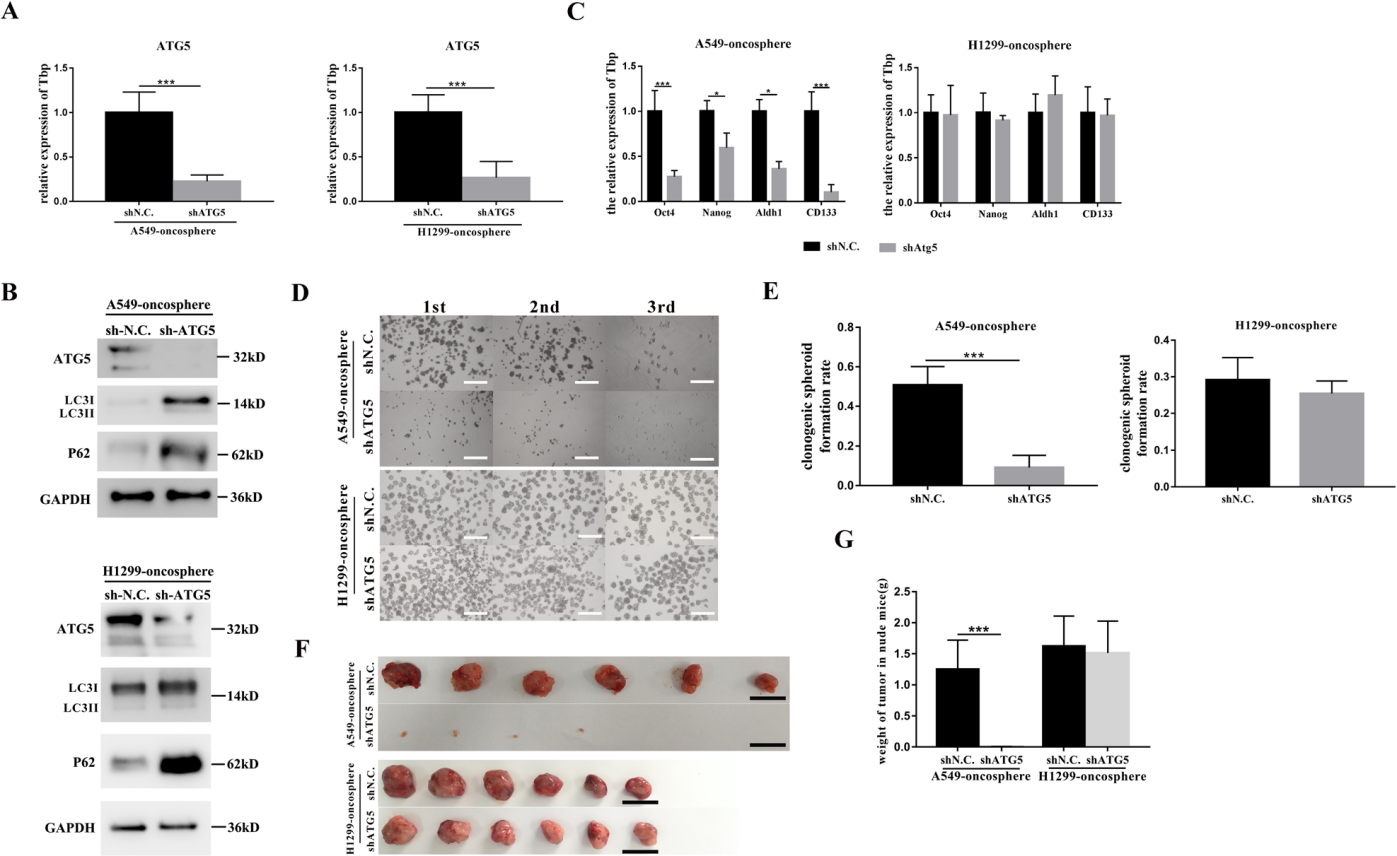
The stemness of A549-oncosphere, and not that of the H1299-oncosphere, is inhibited upon ATG5 silencing. **A** Analysis of ATG5 mRNA expression (**A** Tbp was used as a reference control. ***p < 0.01.) and protein expression (**B** GAPDH was used as a reference control) in A549-oncosphere and H1299-oncosphere, shN.C. was the negative control. **C** Analysis of mRNA expression of stem cell markers Oct4, Nanog, Aldh1, and CD133 upon ATG5 silencing in A549-oncosphere and H1299-oncosphere cells; Tbp was used as a reference control. *p < 0.05, ***p < 0.01. Colony formation assay (**D** bar = 120 µm) and single-cell cloning assay, 1st means single-cell suspensions were plated at 1000 cells/well, 2nd means spheroid cultures were then collected from 1st and single-cell suspensions were prepared for setting up the second round of the assay at 1000 cells/well, and so forth. The 3rd means 1000 cells came from the 2nd (**E** ***p < 0.01.) using A549-oncosphere and H1299-oncosphere in which ATG5 was silenced. **F**, **G** Subcutaneous tumors excised (**F** images; **G** tumor weight, ***:***p < 0.01:*p* < 0.01) from nude mice receiving A549-oncosphere-shATG5, H1299-oncosphere-shN.C. and H1299-oncosphere-shATG5 cells. Bar = 1 cm.

To confirm that the stemness of A549-oncosphere and H1299-oncosphere is differentially regulated by autophagy, ATG5, the regulator of the autophagy initiation, was effectively knocked down by lentivirus-mediated gene silencing in both CSC cell lines (Fig. 3A, B). In vitro assessment of the stemness showed that while prevention of autophagy by ATG5 silencing significantly reduced the stemness of A549-oncosphere, it had no significant effect on the H1299-oncosphere (Fig. 3C–E). Tumor formation in nude mice was induced by injecting 10^3^ cells of A549-oncosphere-shN.C., A549-oncosphere-shATG5, H1299-oncosphere-shN.C., or H1299-oncosphere-shATG5 subcutaneously into the hind legs. Tumor growth was evaluated on day 21 post inoculation. Although tumor volume and weight of mice receiving A549-oncosphere-shATG5 were significantly reduced, silencing of ATG5 produced no significant effect on tumor growth in mice receiving H1299-oncosphere-shATG5 (Fig. 3F, G). These observations indicated that: (1) the basal level of stemness and autophagy activity are enhanced in the A549- and H1299-oncospheres compared with their respective parental cell line (Fig. 2A–E); (2) although the stemness of A549-oncosphere is subjected to autophagy regulation, it is independent of alterations in autophagy in the H1299-oncosphere (Fig. 2H–J, Fig. 3).

### Regulation of the stemness of human lung CSCs is TP53-dependent

**Fig. 4:**
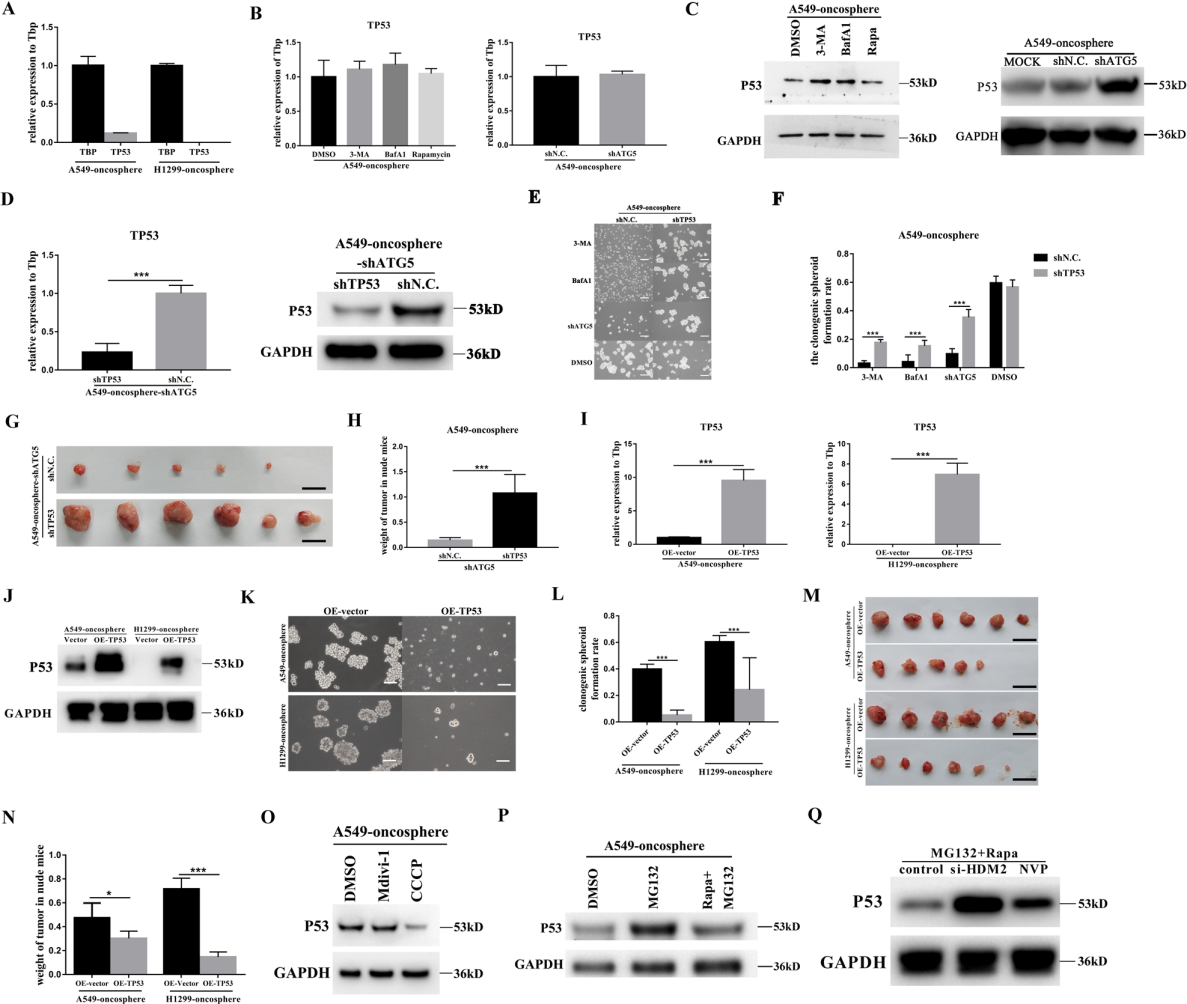
Augmentation of the stemness of human lung CSCs by autophagy is dependent on degradation of ubiquitinated p53. **A** Analysis of mRNA expression of TP53 in A549-oncosphere, and H1299-oncosphere; Tbp was used as a reference control. **B** Analysis of mRNA expression (**B** Tbp was used as a reference control) and protein level of p53 (**C** GAPDH was used as a reference control) in A549-oncosphere treated with DMSO, 3-MA, BafA1, rapa, and knockdown of ATG5. **D** Analysis of mRNA expression and protein levels of p53 in A549-oncosphere-shATG5 after siRNA knockdown of TP53 (A549-oncosphere-shATG5-shTP53); Tbp and GAPDH were used as a reference controls, ***p < 0.01. Colony formation assay (**E** bar = 120 µm) and single-cell cloning assay (**F** ***p < 0.01) using A549-oncosphere-shATG5 and H1299-oncosphere-shATG5 in which ATG5 cells treated with 3-MA, BafA1 followed knockdown of TP53, DMSO was the control group**. G**, **H** Subcutaneous tumors excised (**G**, images; **H**: tumor weight, *p* < 0.01) from nude mice receiving A549-oncosphere-shATG5-shTP53, A549-oncosphere-shATG5-shN.C cells. Bar = 1 cm. **I**, **J** Analysis of mRNA expression and protein levels of p53 in A549-oncosphere and H1299-oncosphere after overexpression of TP53 (A549-oncosphere-OE-TP53 and H1299-oncosphere-OE-TP53); Tbp and GAPDH were used as reference controls, ***p < 0.01. Colony formation assay (**K** bar = 120 µm) and single-cell cloning assay (**L** ***p < 0.01.) using A549-oncosphere cells overexpressing TP53 (A549-oncosphere-OE-TP53 and H1299-oncosphere-OE-TP53). **M**, **N** Subcutaneous tumors excised (**M**, images; **N**: tumor weight, ***p < 0.01) from nude mice receiving A549-oncosphere cells overexpressing TP53 (A549-oncosphere-OE-TP53 and H1299-oncosphere-OE-TP53). Bar = 1 cm. **O**, **P** Analysis of protein levels of p53 in A549-oncosphere treated with DMSO, Mdivi-1, CCCP **O**, MG132 and MG132 in combination with rapa (**P**) by WB; GAPDH was used as a reference control. **Q** Analysis of protein level of p53 in A549-oncosphere upon HDM2 silencing (di-HDM2) or NVP, followed by treatment with MG132 in combination with rapa; GAPDH was used as a reference control. The concentration of 3-MA, BafA1, rapa, Mdivi-1, CCCP, MG132, and NVP was 3 mM, 100 nM, 5 µM, 75 µM, 50 µM, 40 µM, and 300 nM, respectively, and cells used in this figure were treated for 24 h.

The expression types of TP53 were different in A549 and H1299 such that A549 expresses the wild-type TP53, and TP53 is deleted in H1299 cell line^40^. We first verified TP53 expression in the A549-oncosphere and its absence in H1299-oncosphere cells by q-PCR (Fig. 4A). TP53 mRNA expression in A549-oncosphere cells was not altered when autophagy was either inhibited by 3-MA, BafA1, and ATG5 silencing, or stimulated by rapa (Fig. 4B). However, the level of the protein p53 was increased when autophagy was inhibited (Fig. 4C). These results indicate that TP53 is subjected to translational regulation by autophagy in lung CSC cells that express the wild-type TP53.

Elevation of p53 protein expression was seen in A549-oncosphere cells when stemness was inhibited upon autophagy inhibition (A549-oncosphere-shATG5) (Fig. 4C). Thus, we effectively silenced TP53 expression in A549-oncosphere cells in which ATG5 was silenced and we observed decreased p53 protein expression (the A549-oncosphere-shATG5 cells, Fig. 4D). We followed up with a rescue experiment. TP53 silencing prevented autophagy inhibition (shATG5) induced elevation of p53 protein expression and attenuation of stemness in the A549-oncosphere both in vitro and in vivo (Fig. 4E–H). These data show that regulation of stemness in lung CSCs by autophagy requires functional p53.

To investigate the mechanism of TP53 in regulation of lung CSC stemness, TP53 was stably overexpressed in A549- and H1299-oncosphere cells via lentivirus (Fig. 4I, J). TP53 overexpression significantly reduced the colony formation and tumor formation in A549- and H1299-oncospheres in vitro and in vivo (Fig. 4K–N). Further studies are required to determine whether these effects are the result of inhibition of stemness via TP53 overexpression or due to the overexpression of wild-type TP53 inducing cell killing.

### Autophagy regulates stemness of human lung CSCs by the degradation of ubiquitinated p53

A recent report demonstrated that p53 anchored on the mitochondria could be degraded by mitophagy^18^. As inhibition of autophagy did not change the transcription level of TP53 (Fig. 4B), but increased TP53 protein expression in A549-oncosphere cells proved by P53/GAPDH ratio (Figs. 4C and S1D), autophagy appears to regulate TP53 at the translational level. To investigate whether mitophagy has a role in p53 degradation in A549-oncosphere, cells were treated with Mdivi-1, the mitophagy inhibitor, or CCCP, the mitophagy inducer. Although activation of mitophagy by CCCP lead to a reduction of the p53 protein level, inhibition of mitophagy by Mdivi-1 had no significant effect in which the efficiency of Mdivi-1 had been detected through the protein level of COXIV, TOM20, and TIM23 (Figs. 4O and S2D). Therefore, p53 degradation in A549-oncosphere is not achieved by mitophagy.

The ubiquitin-proteasome system (UPS) is the most important mechanism for p53 degradation^41^. Few studies show that autophagy can capture ubiquitinated proteins for degradation^17^. Thus, we examined whether autophagy could degrade ubiquitinated p53 in A549-oncosphere CSCs. Treatment of A549-oncosphere with proteasome inhibitor MG132 lead to an increase of p53 protein levels and such increases could be blunted by stimulation of autophagy with rapa (Fig. 4P). In addition, MG132 treatment also decreased the A549-oncosphere single-cell cloning efficiency and tumor formation (Fig. S2A, B). To evaluate whether autophagy can degrade ubiquitinated p53, HDM2, the E3 ubiquitin ligase of p53, was effectively knocked down by siRNA (Fig. S2C), or inhibited by its inhibitor NVP-CGM097. P53 levels were increased upon HDM2 siRNA knockdown and were decreased when proteasome inhibitor, MG132, and autophagy inducer, rapa, were added to the cells (Figs. 4P and S2E). At last, there were the co-located of P53(red) with LC3(green) and LAMP1(purple) by IF in the presence of BafA1 (Fig. S2F). Therefore, autophagy was one way of degrading cytosolic ubiquitinated p53, thus allowing autophagy augmentation of the stemness of lung CSCs.

### Autophagy-p53-Zeb1 axis regulates the stemness of human lung CSCs

**Fig. 5:**
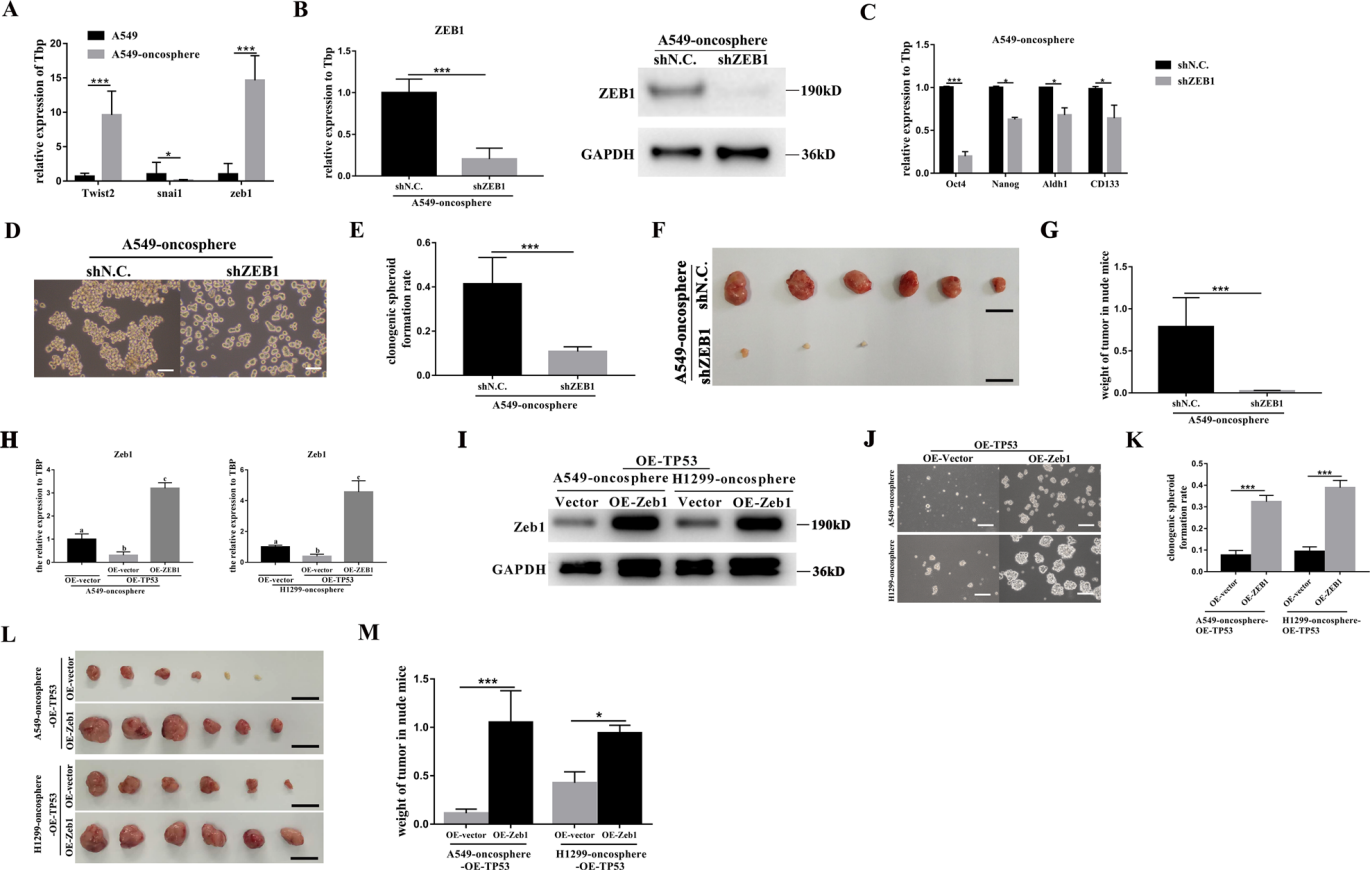
Zeb1 is the downstream effector of p53 in regulating the stemness of human lung CSCs. **A** Analysis of mRNA expression of Twist2, Snail1, and Zeb1 in A549 and A549-oncosphere; Tbp was used as a reference control, *p < 0.05, ***p < 0.01. **B** Analysis of mRNA expression and protein level of Zeb1 in A549-oncosphere after TP53 silencing (A549-oncosphere-shZEb1); Tbp and GAPDH were used as a references controls, ***p < 0.01. **C** Analysis of mRNA expression of Oct4, Nanog, Aldh1, and CD133 upon Zeb1 silencing (A549-oncosphere-shZEB1); Tbp was used as a reference control, *p < 0.05, ***p < 0.01. Colony formation assay (**D** bar = 120 µm) and single-cell cloning assay (**E** ***p < 0.01.) using A549-oncosphere cells in which Zeb1 was silenced (A549-oncosphere-shZEB1). **F**, **G** Analysis of the tumors obtained from nude mice receiving A549-oncosphere-shN.C. and A549-oncosphere-shZEB1 cells, ***p < 0.01. Bar = 1 cm. **H** Analysis of mRNA expression of Zeb1 in A549-oncosphere-OE-TP53 and H1299-oncosphere-OE-TP53 after overexpression of Zeb1, the control groups were A549-oncosphere-OE-vector and H1299-oncosphere-OE-vector, respectively. Tbp was used as a reference control, different superscripts represent significant differences, *p* < 0.05. **I** Analysis of protein level of Zeb1 in A549-oncosphere-OE-TP53 and H1299-oncosphere-OE-TP53 after overexpression of Zeb1, the control groups were A549-oncosphere-OE-vector and H1299-oncosphere-OE-vector, respectively. GAPDH was used as a reference control. Colony formation assay (**J** bar = 120 µm) and single-cell cloning assay (**K** ***p < 0.01.) using A549-oncosphere-OE-TP53 and H1299-oncosphere-OE-TP53 overexpressing Zeb1 (A549-oncosphere-OE-TP53-OE-ZEB1 and H1299-oncosphere-OE-TP53-OE-ZEB1). **L**, **M** Subcutaneous tumors exercised (**L**, images; **M**: tumor weight, *p < 0.05, ***p < 0.01) from nude mice receiving A549-oncosphere-OE-TP53 and H1299-oncosphere-OE-TP53 overexpressing Zeb1 (A549-oncosphere-OE-TP53-OE-ZEB1 and H1299-oncosphere-OE-TP53-OE-ZEB1). Bar = 1 cm.

As previously mentioned, Zeb1, a downstream effector of TP53 could regulate the stemness of the mouse lung CSCs^26,29,34^. To explore the molecular relationship between p53 and Zeb1 in the regulation of stemness of human lung CSCs, Zeb1 was knocked down in A549-oncosphere, such that the expression of Zeb1 and Twist2 were significantly higher than that in A549 parental cells (Fig. 5A). Effective Zeb1 silencing was confirmed by q-PCR and WB (Fig. 5B). Silencing of Zeb1 expression lead to significant inhibition of the stemness of A549-oncosphere cells both in vitro and in vivo (Fig. 5C–G).

As p53 negatively regulates Zeb1 transcription, a rescue experiment was then conducted in which TP53 and Zeb1 was double overexpressed (OE-TP53-OE-Zeb1) in A549-oncosphere and H1299-oncosphere cells (Fig. 5H, I). Zeb1 overexpression reversed the inhibition of TP53 overexpression on the stemness of both A549- and H1299-oncospheres (Fig. 5J, M). These data indicated that Zeb1 is downstream of p53 and the p53-Zeb1 axis regulates the stemness of human lung CSCs.

**Fig. 6:**
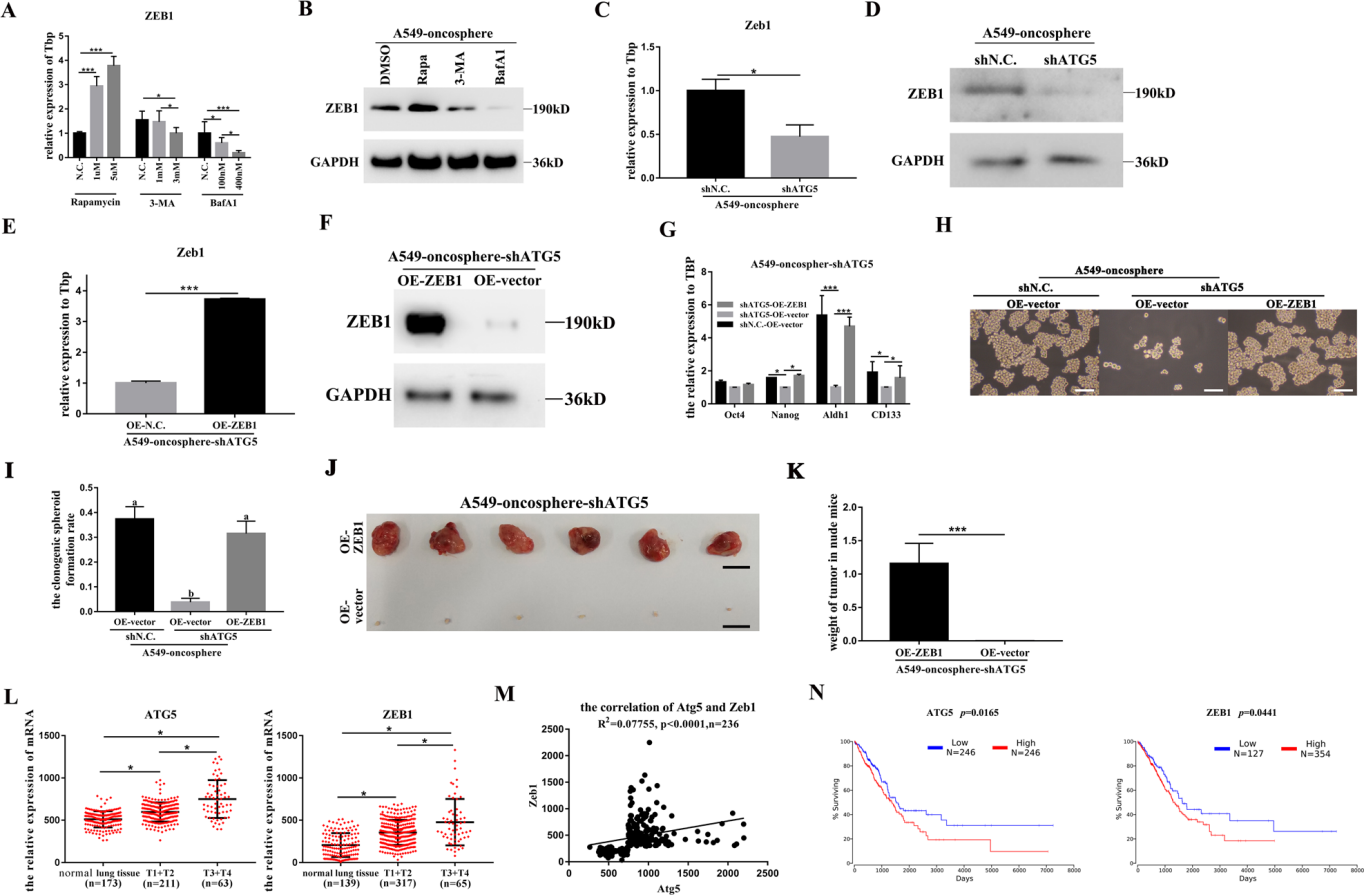
Augmentation of the stemness of human lung CSCs requires cooperation of Zeb1 in A549-oncosphere and analysis of clinical relevance of ATG5 and Zeb1. **A**–**D** Analysis of mRNA expression and protein level of Zeb1 in A549-oncosphere treated by DMSO, rapa, 3-MA, and BafA1 **A**, **B**; and upon silencing of ATG5 **C**, **D**; Tbp and GAPDH were used as reference controls, *p < 0.05. The concentrations of 3-MA, BafA1, and rapa were 3 mM, 100 nM, and 5 µM, respectively, and cells in Fig. 6B were treated for 24 h. **E**, **F** Analysis of mRNA expression and protein level of Zeb1 in A549-oncosphere-shATG5 after overexpression of Zeb1 (A549-oncosphere-shATG5-OE-ZEB1); Tbp and GAPDH were used as reference controls, ***p < 0.01. **G** Analysis of mRNA expression of Oct4, Nanog, Aldh1, and CD133 in A549-oncosphere-shN.C.-OE-vector, A549-oncosphere-shATG5-OE-vector, and A549-oncosphere-shATG5-OE-ZEB1; Tbp was used as a reference control, *p < 0.05, ***p < 0.01. Colony formation assay (**H** bar = 120 µm) and single-cell cloning assay (**I** different superscripts represent significant differences, *p* < 0.05), using A549-oncosphere-shN.C.-OE-vector, A549-oncosphere-shATG5-OE-vector, and A549-oncosphere-shATG5-OE-ZEB1 cells. **J**, **K** Analysis of the tumors obtained from mice receiving A549-oncosphere-shATG5-OE-vector and A549-oncosphere-shATG5-OE-ZEB1, ***p < 0.01. Bar = 1 cm. **L** mRNA expression of ATG5 and ZEB1 in normal tissue and different stages of human lung adenocarcinoma using TCGA data sets, *p < 0.05. **M** Analysis of the correlation of ATG5 and ZEB1 in human lung adenocarcinoma using TCGA datasets; R^2^ was the correlation coefficient. **N** Survival analysis of ATG5 and ZEB1 in human lung adenocarcinoma using TCGA datasets by OncoLnc.

We then explored the relationship between autophagy and Zeb1 in the regulation of the stemness of lung CSCs. We observed that mRNA and protein levels of Zeb1 were subjected to positive regulation by autophagy (Fig. 6A–D). We also detected the expression of Zeb1 by q-PCR and WB with the additions of 3-MA, BafA1, and rapa, which showed that rapa could decrease the mRNA and protein levels of Zeb1 in the H1299-oncosphere (Fig. S3A, B). A rescue experiment was conducted in which overexpression of Zeb1 (OE-Zeb1) (Fig. 6E, F) prevented ATG5 knockdown-induced inhibition of stemness in the A549-oncosphere (A549-oncosphere-shATG5-OE-Zeb1) both in vitro and in vivo (Fig. 6G–K). These results demonstrate that Zeb1 is a downstream mediator of autophagy in the regulation of stemness of the A549-oncosphere.

### Clinical significance of autophagy and Zeb1 in human lung cancer

The clinical relevance of the relationship between autophagy and Zeb1 was evaluated in human lung adenocarcinoma using clinical TCGA datasets. The expression of ATG5 and Zeb1 were both positively correlated with the stage of human lung adenocarcinoma (Fig. 6L). In addition, the expression of ATG5 was also positively correlated with the expression of Zeb1 in human lung adenocarcinoma (Fig. 6M). OncoLnc analysis shows that elevated expression of ATG5 and Zeb1 conferred poorer survival rate (Fig. 6N). These findings suggest that high autophagy activity and high Zeb1 expression are valid prognostic markers for poor prognosis in human lung cancer.

## Discussion

In the literature, both the pro- and anti-oncogenic activities of autophagy have been reported and are context-dependent^7,25,42^. Augmentation of CSC stemness by autophagy has received increased attention^3–5^, yet mechanistic characterization is lacking^8^. In the present study, by generating stable human lung CSC cell lines with the wild-type TP53 (A549) and cell lines in which TP53 is deleted (H1229), we show, for the first time, that autophagy augments the stemness of lung CSCs by degrading ubiquitinated p53. Furthermore, Zeb1 mediates p53 regulation of CSC self-renewal. Moreover, TCGA data mining and analysis show that Atg5 and Zeb1 are poor prognostic markers of lung cancer. This study has made the following novel findings to elucidate a new CSC-based mechanism that underlies the oncogenic activity of autophagy and the tumor suppressor activity of p53 in cancer via regulation of the self-renewal of cancer-initiating cells, the CSCs (Fig. S4).

First, augmentation of stemness by autophagy can be realized by degrading ubiquitinated p53, thus relieving the tumor suppressor activity of p53. Previous reports have shown that p53 functions upstream of autophagy and regulates autophagy depending on its intracellular localization^17,43^. When p53 is in the nucleus, it promotes autophagy by transcriptional activation of genes that are critical for autophagy. However, cytosolic localized p53 inhibits autophagy through AMPK and mTOR (Fig. S1C)^17,43^. Before our study, few studies have shown that autophagy can regulate the expression of p53 though mechanisms not yet clarified^17,18^. Here, we show p53 can also function downstream of autophagy (Fig. 3) and is subjected to autophagy regulation at the post-translational level.

The main pathway of p53 degradation is the UPS^41,44^. In addition, mitophagy degradation of p53 has been demonstrated in hepatic CSCs^18^. In this study, we ruled out the mitophagy mechanism of p53 degradation in lung CSCs (Fig. 4). Previous studies have reported that selective autophagy in which p62, NBR1, NDP52, HDAC6, and ALFY will bind ubiquitin through their ubiquitin-associated domain and deliver ubiquitinated proteins to the phagophore^45,46^. In our study, the protein level of p53 was also increased significantly when the proteasome was inhibited by MG132, whereas it was reduced when autophagy was induced. Furthermore, inhibition of HDM2, the p53 ubiquitination-specific regulator, increased p53 expression when the proteasome was inhibited by MG132 and autophagy was induced (Fig. 4). Collectively, these observations indicate that ubiquitinated p53 in the cytoplasm can be degraded by autophagy in human lung CSCs. Future investigations are required to elucidate how, and which autophagy receptor can selectively capture the ubiquitinated p53 in human lung CSCs.

Second, we have characterized the axis of p53-Zeb1 as the mechanism underlying autophagy augmentation of stemness inhuman lung CSCs. Enhanced stemness of human lung CSCs by Zeb1 is consistent with our previous study conducted using the mouse Lewis lung cancer cells^34^. Molecular ordering experiments have placed Zeb1 to be downstream of p53 (Fig. 5) and of autophagy (Fig. 6). However, elucidation of the mechanisms underlying p53 regulation of Zeb1 is beyond the scope of this study and merits future investigation. Reports have shown that inhibition of Zeb1 by p53 can be mediated by miR-200 during tumor metastasis^13,35^, which was also validated in our study (Fig. S3C). In this study, we show that autophagy activity increases the expression of Zeb1 (Fig. 6), consistent with literature reports on autophagy augmentation of cancer cell stemness by promoting EMT^47–49^, where Zeb1 also plays a role.

Finally, the autophagy-p53-Zeb1 axis can be explored clinically for lung cancer prognosis. High autophagy activity (high ATG5 expression) and high Zeb1 expression are correlated and associated with poor clinical outcomes in human lung cancer. The most important clinically relevant finding derived from our study is that TP53 status, i.e., whether the wild-type is expressed, determines the susceptibility of lung CSCs to be regulated by autophagy. In lung CSCs where TP53 is deleted or is functionally mutated, self-renewal is independent of alterations in autophagy activity.

In summary, this study has elucidated a new mechanism underlying the oncogenic activity of autophagy and the tumor suppressor activity of p53 in cancer, via the exploitation of the autophagy-p53-Zeb1 axis by CSCs for self-renewal.

## Supplementary information

supplemental Figures

## Supplementary information

**Supplementary Information** The online version contains supplementary material available a (10.1038/s41419-021-03392-6).
