# Supplementary material for: Autophagy augments the self-renewal of lung cancer stem cells by the degradation of ubiquitinated p53
Source: Cell Death Dis. 2021 Jan 19;12(1):98. doi: 10.1038/s41419-021-03392-6 (PMC7815724; doi:10.1038/s41419-021-03392-6)
Supplement: Supplementary file 1 — supplemental Figures [file 41419_2021_3392_MOESM1_ESM.docx]

**
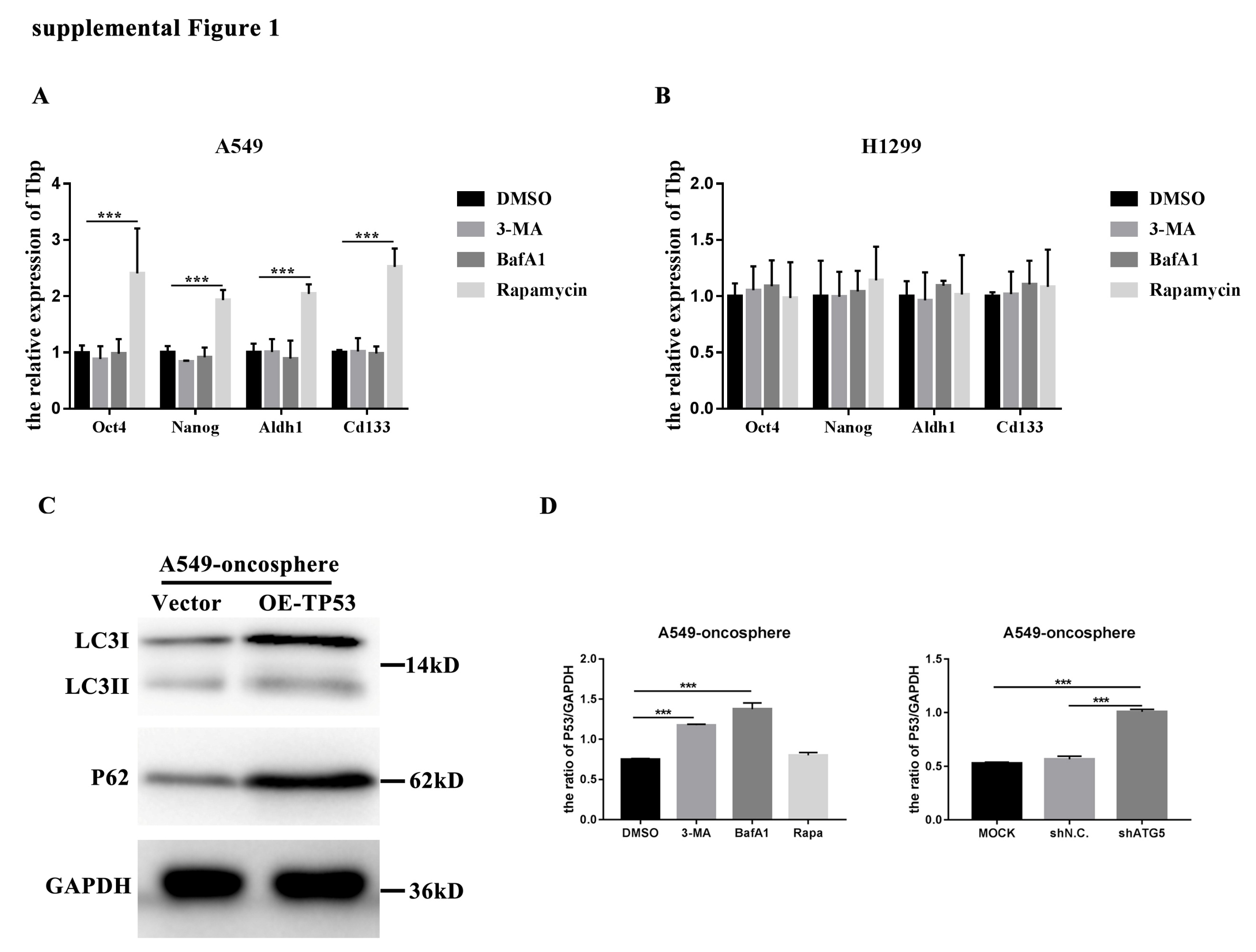
**

**Supplemental Figure 1. A** and **B.** mRNA expression of Oct4, Nanog, Aldh1, and CD133 in A549 and H1299 treated with DMSO, 3-MA, BafA1, and rapamycin. Tbp was used as a reference control. ***p<0.05. **C.** Protein expression of LC3 and P62 in A549-oncosphere-OE-vector and A549-oncosphere-OE-Tp53, GAPDH was used as a reference control. **D.** Western blot analysis of P53/GAPDH ratio in A549-oncosphere treated with DMSO, 3-MA, BafA1, rapamycin, and knockdown of ATG5 by ImageJ, ***p<0.05.


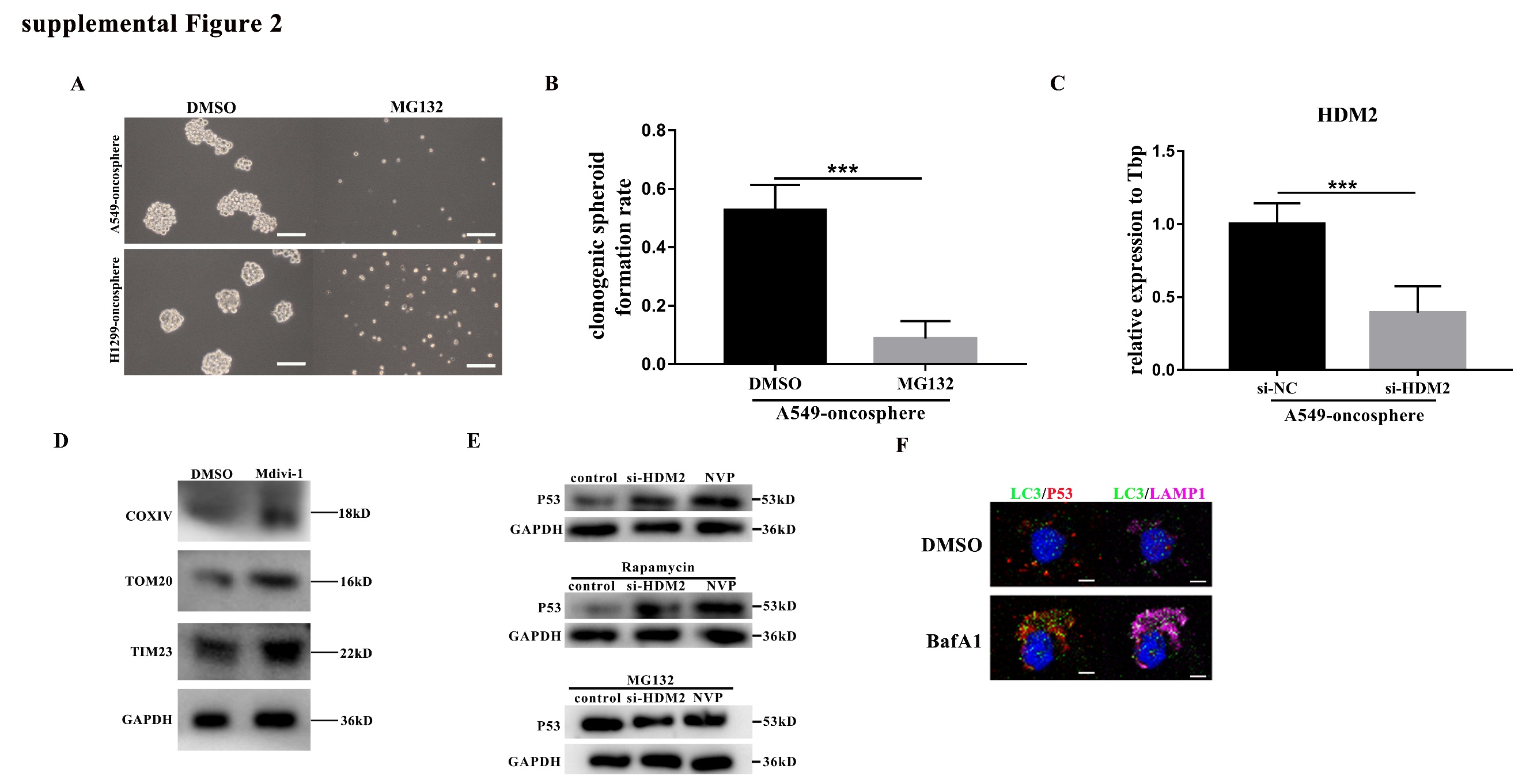


**Supplemental Figure 2. A.** Colony formation assay using A549-oncosphere and H1299-oncosphere treated with DMSO and MG132, bar=120 um. **B.** Analysis of single-cell cloning assay using A549-oncosphere-sh-ATG5-OE-vector and A549-oncosphere-shATG5-OE-ZEB1 cells, ***p<0.01. **C**. Analysis of mRNA expression of HDM2 in A549-oncosphere after siRNA silencing of HDM2, Tbp was used as a reference control, ***p<0.01. **D.** Protein expression of COXIV, TOM20, and TIM23 in A549-oncosphere added with DMSO and Mdivi-1, GAPDH was used as a reference control. **E.** Protein expression of P53 in A549-oncosphere added with si-HDM2 and NVP in the presence of MG132 or rapamycin, control group was added with DMSO, GAPDH was used as a reference control. **F.** Tthe co-located of P53(red) with LC3(green) and LAMP1(purple) by immunofluorescence in the presence or absence of BafA1, white bar=30 um.


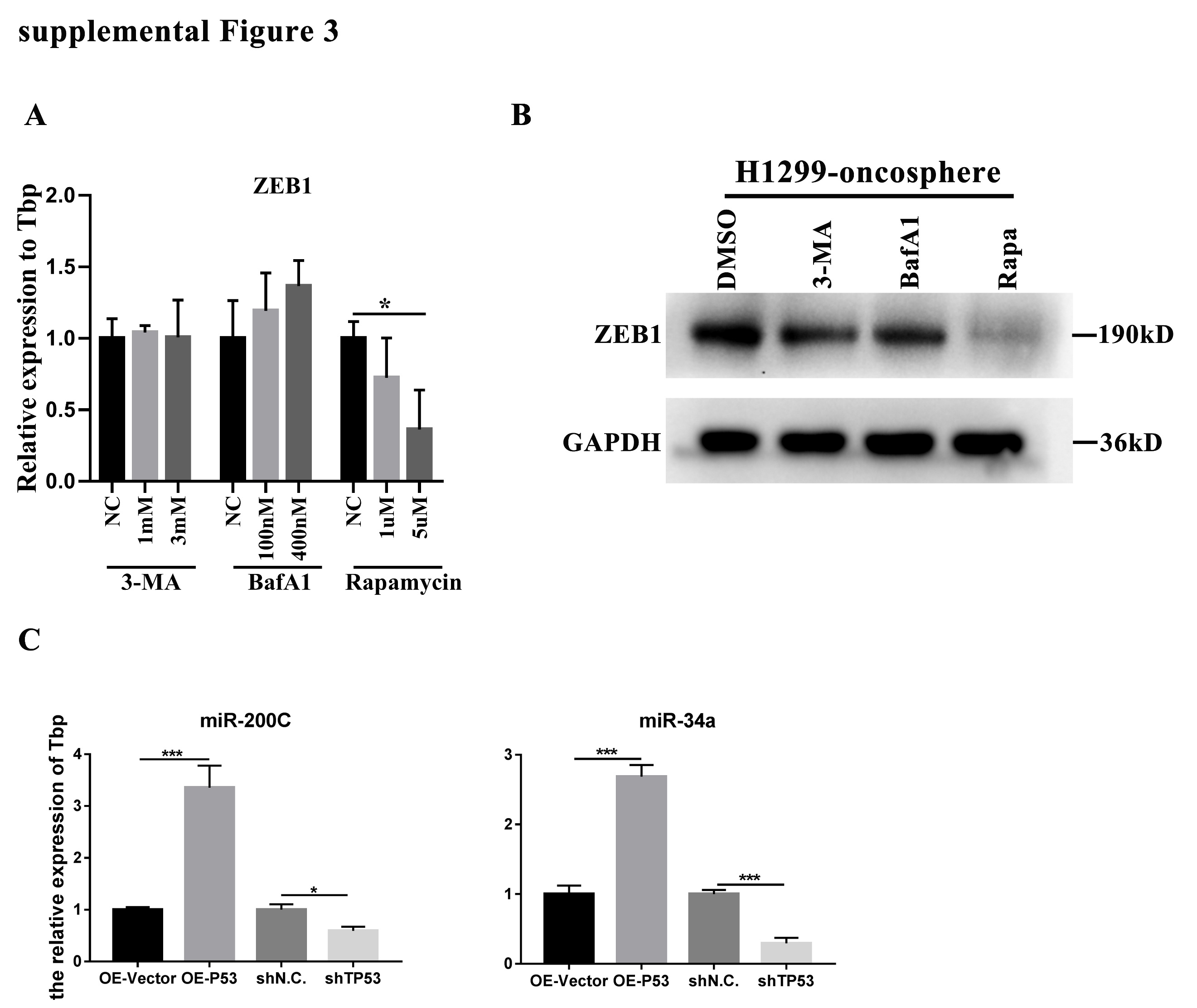


**Supplemental Figure 3. A** and **B.** Analysis of mRNA expression and protein levels of Zeb1 in A549-oncosphere treated by DMSO, rapamycin, 3-MA, BafA1. Tbp and GAPDH were used as reference controls, *p<0.05. C. Analysis of miRNA expression levels of miR-200C and miR-34a in A549-oncosphere infected by OE-Vector, OE-P53, shN.C., and shTP53.


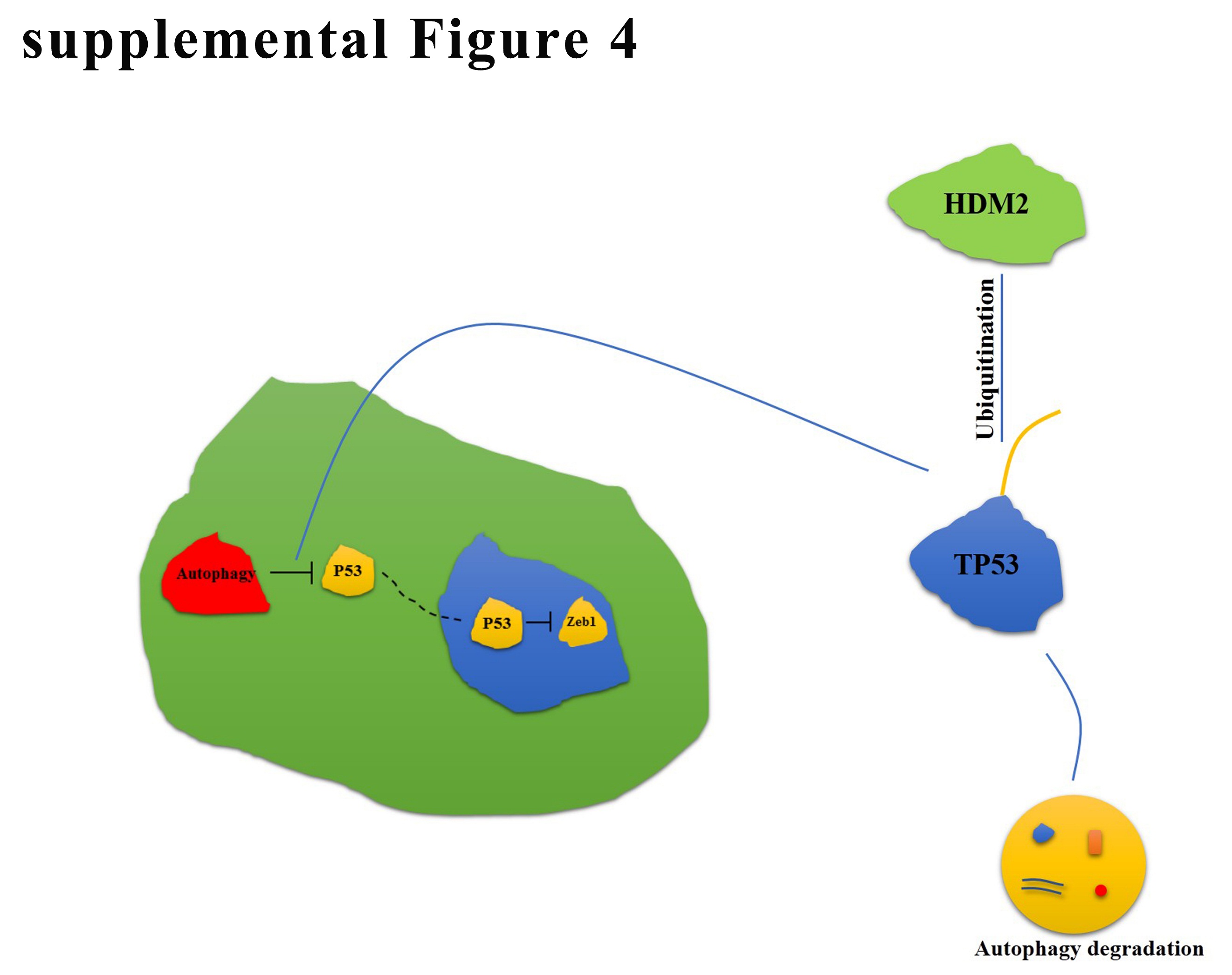


**Supplemental Figure 4.** Schematic summary of the mechanisms underlying the autophagy-TP53-Zeb1 axis in the regulation of the stemness of lung CSCs.
